# Pia Mater‐Penetrable Lipopolymer Nanoparticles for Gliocyte‐Targeted IL‐10 mRNA Therapy Alleviate Paclitaxel‐Induced Peripheral Neuropathy

**DOI:** 10.1002/advs.202500362

**Published:** 2025-05-13

**Authors:** Xinrou Lin, Lingyu Wei, Xiangpen Li, Ling Zeng, Yingsen Tang, Hongjin Wang, Hengjian Lu, Chenguang Li, Hongxuan Wang, Jinjin Chen, Ying Peng

**Affiliations:** ^1^ Department of Neurology Sun Yat‐Sen Memorial Hospital Sun Yat‐Sen University Guangzhou 510000 China; ^2^ Nanhai Translational Innovation Center of Precision Immunology Sun Yat‐Sen Memorial Hospital Foshan 528200 China; ^3^ Department of Anesthesiology Sun Yat‐Sen Memorial Hospital Sun Yat‐Sen University Guangzhou 510000 China; ^4^ Guangdong Provincial Key Laboratory of Malignant Tumor Epigenetics and Gene Regulation Sun Yat‐Sen Memorial Hospital Sun Yat‐Sen University Guangzhou 510000 China; ^5^ Shenshan Medical Center Sun Yat‐Sen Memorial Hospital Sun Yat‐Sen University Shanwei 516600 China; ^6^ Department of Urology Sun Yat‐Sen Memorial Hospital Sun Yat‐Sen University Guangzhou 510000 China

**Keywords:** dorsal root ganglion, IL‐10, lipopolymer nanoparticle, mRNA, paclitaxel‐induced peripheral neuropathy

## Abstract

Paclitaxel (PTX) is a commonly used chemotherapeutic agent for treating various solid tumors; however, it often leads to a severe side effect known as paclitaxel‐induced peripheral neuropathy (PIPN), for which effective treatments are limited. Although mRNA therapies have shown promise in addressing central nervous system (CNS) disorders, the successful delivery of mRNA therapeutics to the nervous system is still hindered by many biological barriers. In this study, it is demonstrated that, compared with commercial MC3 lipid nanoparticles (MC3 LNPs), mRNA‐loaded P6CIT‐derived lipopolymer nanoparticles (P6CIT LPNPs), which are delivered via intrathecal injection, achieve effective penetration through the pia mater. More importantly, this P6CIT LPNP demonstrates the ability to achieve highly targeted mRNA transfection in gliocytes within the spinal cord and dorsal root ganglia (DRG), which is essential for the regulation of neuroinflammation. Furthermore, two intrathecal injections of P6CIT LPNPs encapsulating mIL‐10 (P6CIT/mIL‐10) significantly alleviate PIPN by reducing proinflammatory cytokine production, gliocyte activation, and presynaptic NMDA receptor hyperactivity in both male and female mice. This study presents a promising and clinically translatable platform for using mRNA‐loaded LPNPs to treat PIPN.

## Graphical abstract

The P6CIT lipopolymer demonstrates superior transfection efficacy in the spinal cord and DRG, as well as enhanced penetration of the pia mater compared to the MC3 ionizable lipid. Intrathecal delivery of P6CIT LPNPs enables effective transfection in the gliocytes in the spinal cord and DRG. Notably, intrathecal administration of P6CIT/mIL‐10 significantly alleviates allodynia in paclitaxel‐induced peripheral neuropathy mice.

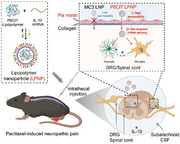

## 1 Introduction

Paclitaxel (PTX) is one of the most widely used chemotherapeutic agents for treating various solid tumors.^[^ ^1^ ^]^ However, approximately 40–60% of patients undergoing PTX treatment develop PTX‐induced peripheral neuropathy (PIPN), for which efficient therapies remain limited.^[^ ^2^ ^]^ The mechanism of PIPN primarily involves the activation of gliocytes in the spinal cord and dorsal root ganglia (DRG), which triggers the onset of neuroinflammation.^[^ ^3^ ^]^ Additionally, elevated presynaptic N‐Methyl‐D‐aspartate receptor (NMDAR) activity in primary sensory neurons within the DRG enhances glutamate release to neurons in the superficial dorsal horn (SDH), thereby amplifying pain signaling in PIPN.^[^ ^4^ ^]^ Previous studies have identified intrathecal delivery of recombinant IL‐10 protein or adeno‐associated virus encapsulated IL‐10 DNA as a promising therapeutic for resolving PIPN.^[^ ^5^ ^]^ Compared with DNA and direct protein delivery, mRNA therapeutics offer several advantages, including no risk of insertional mutagenesis, the facilitation of transient protein expression, and the allowance for natural posttranslational modifications.^[^ ^6^ ^]^ Lipid nanoparticles (LNPs) have emerged as crucial non‐viral delivery platforms for mRNA therapeutics in vivo to enhance mRNA stability and intracellular delivery.^[^ ^7^ ^]^ However, current LNPs for intrathecal IL‐10 mRNA therapy in PIPN still face challenges of crossing multiple biological barriers.^[^ ^8^ ^]^

Similar to the well‐known blood‐brain barrier (BBB) for intravenous drug delivery, the cerebrospinal fluid (CSF)‐brain/spinal cord barrier also exists for intrathecal drug delivery.^[^ ^9^ ^]^ The CSF, which is a clear, watery liquid that circulates within the space between the arachnoid layer and the pia mater, surrounds and protects the brain and spinal cord.^[^ ^10^ ^]^ The pia mater consists of a thin layer of pial cells, and the subpial tissues are rich in collagen fibers.^[^ ^11^ ^]^ Drugs delivered via intrathecal injection must pass through these structures to reach the spinal cord and DRG. Previous studies have shown that large molecules or particles preferentially circulate within the spinal leptomeninges but exhibit limited entry into the underlying parenchyma.^[^ ^11^, ^12^ ^]^ LNPs used for mRNA delivery usually have larger size than biomolecules, making it challenging for LNPs that are administered via intrathecal injection to effectively cross this barrier.

**Figure 1:**
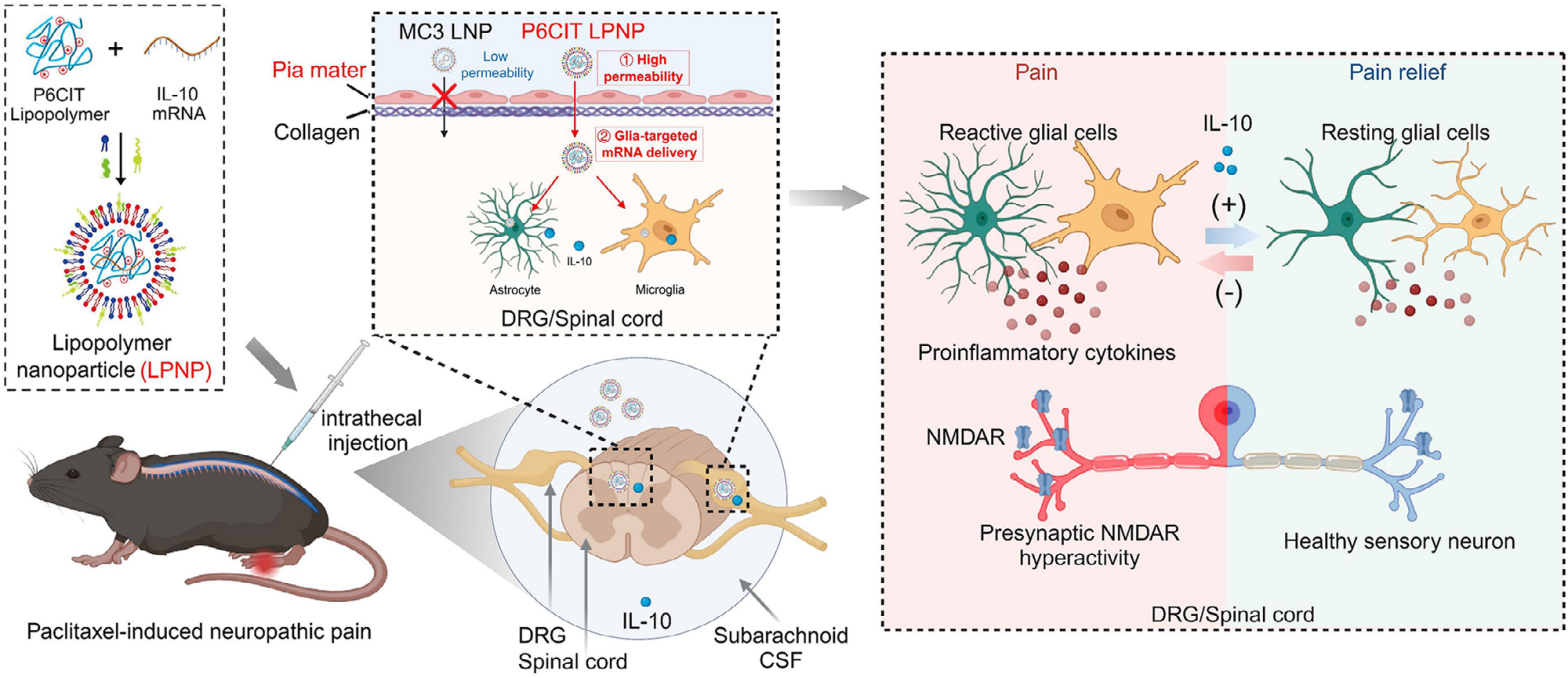
Schematic illustration of the intrathecal delivery of IL‐10 mRNA encapsulated in LPNPs for the treatment of paclitaxel‐induced peripheral neuropathy. The P6CIT lipopolymer demonstrates higher transfection efficacy in the spinal cord and DRG, as well as enhanced penetration of the pia mater and subpial tissues compared to the MC3 ionizable lipid. Intrathecal delivery of P6CIT LPNPs enables successful transfection in the gliocytes in the spinal cord and DRG. P6CIT/mIL‐10 is synthesized and intrathecally injected into mice for the treatment of PIPN. The secreted IL‐10 ameliorates glial cell activation and presynaptic NMDAR hyperactivity, leading to the alleviation of allodynia in PIPN mice.

In this study, we developed lipopolymer nanoparticles (LPNPs) encapsulating IL‐10 mRNA with high pia mater permeability and gliocyte targetability to treat PIPN (**Figure** **1**). First, the branched polyethyleneimine (PEI)‐derived lipopolymer P6CIT was synthesized and formulated into LPNPs, which showed smaller size and increased zeta potential compared with commercial MC3 LNPs. Second, P6CIT LPNPs were demonstrated to exhibit superior mRNA transfection efficacy, CSF stability, and pia mater barrier permeability compared to MC3 LNPs. More importantly, the intrathecal delivery of P6CIT LPNPs majorly expressed in gliocytes including astrocytes and microglia, which is essential for modulating neuroinflammation. Finally, P6CIT LPNPs encapsulating IL‐10 mRNA (P6CIT/mIL‐10), significantly alleviated pain in both female and male PIPN mice. Treatment with P6CIT/mIL‐10 dramatically reduced the paclitaxel‐induced upregulation of IL‐1β, TNFα, GFAP, and IBA1 expression and mitigated presynaptic NMDAR hyperactivity in the lumbar spinal cord and DRG. These findings suggest that LPNPs represent promising platforms for mRNA delivery in spinal cord and DRG‐related disorders, with P6CIT/mIL‐10 potentially serving as an effective therapeutic strategy for PIPN.

## 2 Results

### 2.1 P6CIT LPNPs Enhanced mRNA Transfection and Penetration Compared with MC3 LNPs In Vitro

**Figure 2:**
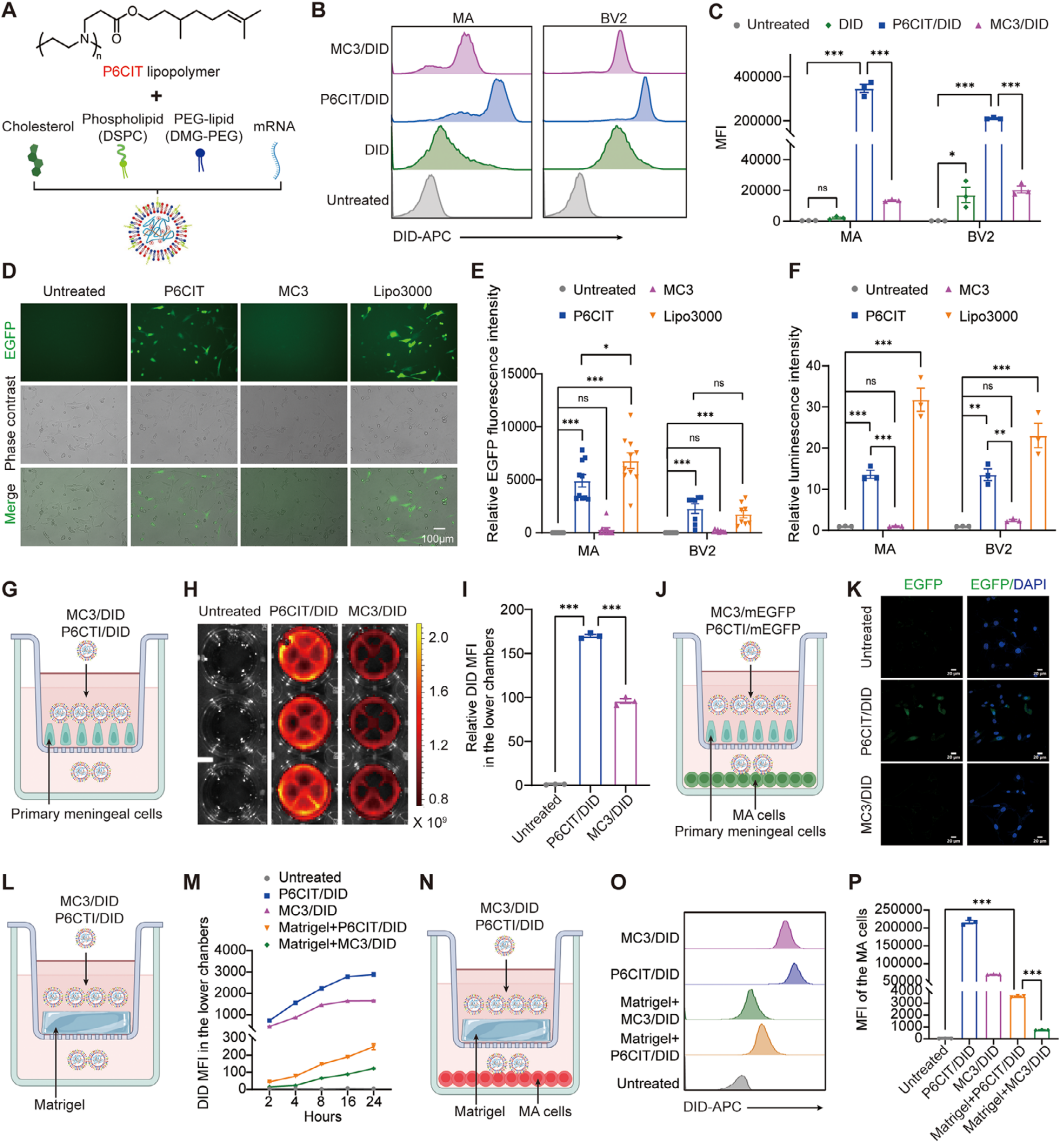
Cellular uptake, transfection efficacy, and CSF‐spinal cord barrier penetration of the mRNA‐loaded MC3 and P6CIT LPNPs in vitro. A) Structure of the P6CIT lipopolymer and synthesis of P6CIT LPNPs. B,C) Cellular uptake of P6CIT/DID in comparison with the uptakes of MC3/DID and free DID was determined using FACS analysis in B) MA and BV2 cells, and quantitative analysis of C) the mean fluorescence intensity (MFI). *n* = 3. D) Representative fluorescence images of MA cells transfected with EGFP mRNA‐loaded LPNPs. Scale bar = 100 µm. E) Quantitative analysis of EGFP expression in MA and BV2 cells transfected with EGFP mRNA‐loaded LPNPs observed via fluorescence microscopy. *n* = 8–10 random fields. F) Quantitative analysis of luciferase activity in MA and BV2 cells transfected with luciferase mRNA‐loaded LPNPs. *n* = 3. G) Schematic of primary meningeal cells seeded in the upper chamber, and incubated with P6CIT/DID or MC3/DID in the transwell assay. H) IVIS imaging showing DID fluorescence in the lower chambers. *n* = 3. I) Relative DID MFI in the lower chambers measured using a microplate reader. *n* = 3. J) Schematic of primary meningeal cells seeded in the upper chamber, and incubated with P6CIT/mEGFP and MC3/mEGFP in the transwell assay. K) Representative fluorescence images of MA cells seeded in the lower chambers. Scale bar = 20 µm. L) Schematic illustrating the Matrigel coating in the upper chamber, with incubation of P6CIT/DID and MC3/DID in the transwell assay. M) DID MFI in the lower chambers at predefined time points. *n* = 3. N) Schematic of Matrigel coating in the upper chamber, with incubation of P6CIT/DID and MC3/DID along with MA cells seeded in the lower chambers in the transwell assay. O,P) Cellular uptake of P6CIT/DID in comparison with MC3/DID in MA cells, analyzed using O) FACS, and P) quantitative analysis of MFI. *n* = 3. Statistical analysis was performed using one‐way ANOVA in (C, E, F, I, and P). The data are expressed as the mean ± SEM; *p < 0.05, **p < 0.01, ***p < 0.001; ns, nonsignificant.

P6CIT LPNPs were synthesized and formulated for optimal mRNA delivery to the spinal cord and DRG via intrathecal injection. The mass ratio of P6CIT, cholesterol, DSPC and PEG‐DMG2000 was 16:12:8:4 (**Figure** **2**A). For comparison, commercial ionizable MC3 LNPs (8.5:2.1:4:1) were also used for mRNA encapsulation. The mass ratio of the ionizable lipids to the mRNA was 10: 1.

Given that astrocytes and microglia modulate the inflammatory state in the CNS, we transfected DID‐labeled nanoparticles into the astrocytic cell line MA and the microglial cell line BV2 to evaluate their cellular uptake abilities. FACS analysis revealed that both MA and BV2 cells exhibited higher uptake of DID‐labeled nanoparticles compared to free DID, suggesting that LPNPs facilitate the cellular internalization of cargos (Figure 2B,C). Moreover, both MA and BV2 cells demonstrated increased uptake of DID‐labeled P6CIT LPNPs (P6CIT/DID) in comparison to DID‐labeled MC3 LNPs (MC3/DID).

mEGFP and mFLuc were used as models to assess the transfection efficacy of mRNAs encapsulated in P6CIT LPNPs and MC3 LNPs in vitro. Lipo3000 served as a positive control for in vitro transfection. Fluorescence images (Figure 2D and Figure S1, Supporting Information) and statistical data (Figure 2E) revealed that P6CIT LPNPs demonstrated superior transfection performance of mEGFP compared to MC3 LNPs. In MA cells, P6CIT LPNPs showed lower gene expression of EGFP mRNA than Lipo3000, while in BV2 cells, the difference was not statistically significant (Figure 2D,E and Figure S1, Supporting Information). For mFLuc‐loaded nanoparticles, P6CIT LPNPs achieved higher transfection efficacy than MC3 LNPs in both MA and BV2 cells, although the efficiency was lower than that of Lipo3000 (Figure 2F).

We then conducted transwell assays to investigate the penetration of LPNPs across the pia mater and subpial tissues in vitro. The upper chamber was seeded with primary meningeal cells to mimic the pia mater and the lower chamber was seeded with MA cells to mimic glia in the spinal cord (Figure 2G,J). First, P6CIT LPNPs were labeled with DID. As shown in Figure 2H,I, a higher fluorescence intensity of DID in the medium of the lower chamber was observed in the P6CIT/DID group compared to the MC3/DID group. Second, the mRNA transfection by P6CIT LPNPs was evaluated by the model EGFP mRNA. The fluorescence of EGFP in the MA cells seeded in the lower chamber was also higher in the P6CIT/mEGFP group compared to the MC3/mEGFP group (Figure 2K). Additionally, the bottom of the upper chamber was coated with Matrigel to simulate the collagens located within subpial tissues (Figure 2L,N). At each predefined time point, the DID MFI in the lower chamber was also higher in the P6CIT/DID group compared to the MC3/DID group, both with and without Matrigel coating in the upper chamber (Figure 2M). The fluorescence of DID in the MA cells seeded in the lower chamber was also higher in the P6CIT/DID group compared to the MC3/DID group, with or without Matrigel coating in the upper chamber (Figure 2P).

### 2.2 P6CIT LPNPs Exhibited Pia Mater‐Penetrable and Glia‐Targeted mRNA Delivery In Vivo

**Figure 3:**
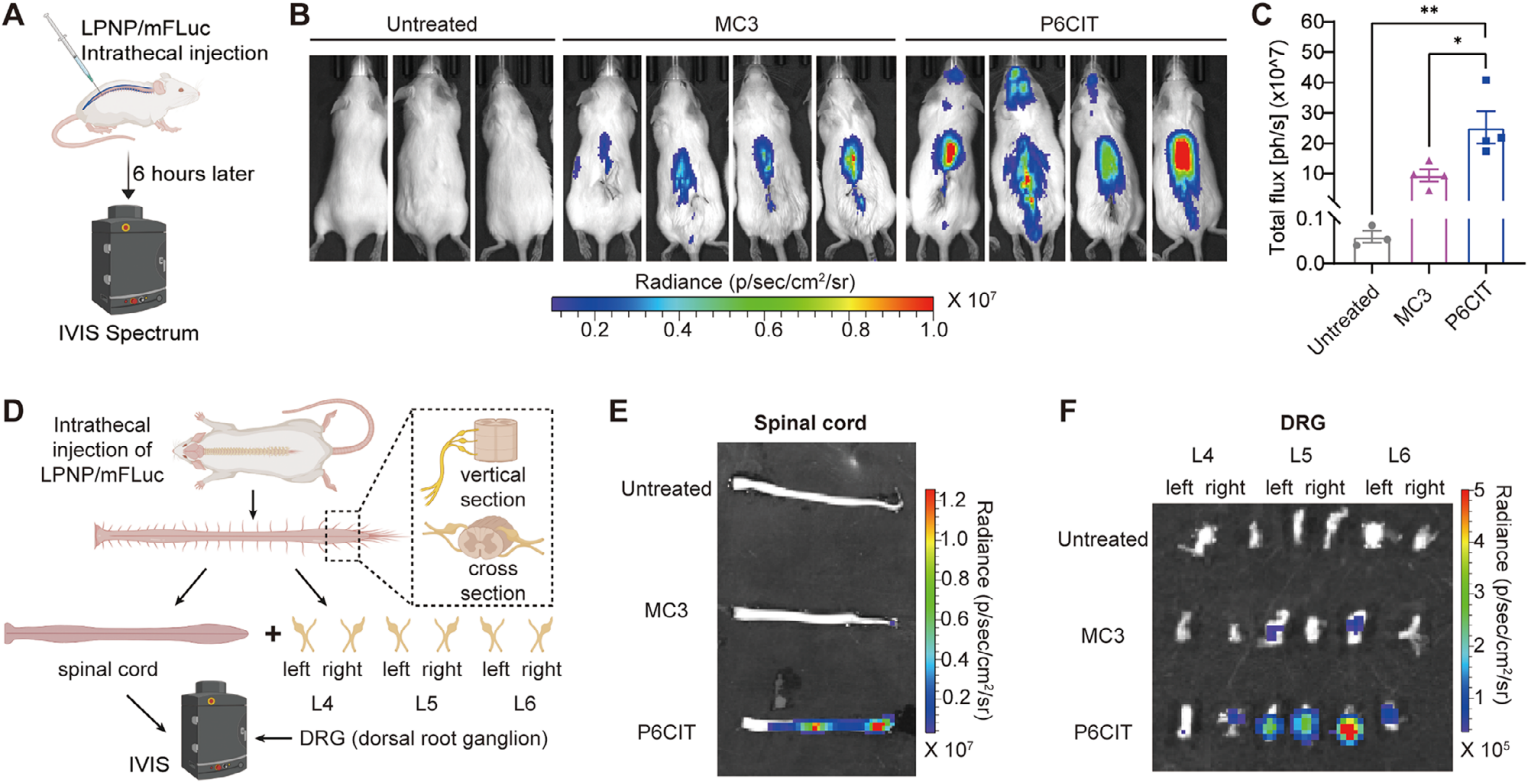
Transfection efficiency in the spinal cord and DRG of ICR mice following the intrathecal injection of mFLuc‐loaded LPNPs. A) Schematic demonstrating LPNP screening in ICR mice via intrathecal injection. B) IVIS images showing luciferase expression in MC3 LPNP‐treated mice and P6CIT LPNP‐treated mice compared with that in untreated mice, C) as well as the corresponding statistical data. *n* = 3–4. D) Anatomical atlas of the spinal cord and DRG, and tissue collection procedure. E,F) IVIS images of E) excised spinal cord and F) DRG from ICR mice treated with MC3 LNPs or P6CIT LPNPs compared with those from untreated controls. Statistical analysis was performed using one‐way ANOVA in (C). The data are expressed as the mean ± SEM; *p < 0.05, **p < 0.01, ***p < 0.001; ns, nonsignificant.

To confirm the in vivo efficiency of P6CIT LPNPs, mFLuc was encapsulated into LPNPs and intrathecally injected into ICR mice. Six hours post‐injection, the mice received an intraperitoneal injection of D‐luciferin sodium, and bioluminescent signals were assessed using an IVIS system (**Figure** **3**A). The mFLuc‐loaded P6CIT LPNPs (P6CIT/mFLuc) showed over two‐fold higher gene expression in comparison to mFLuc‐loaded MC3 LNPs (MC3/mFLuc) (Figure 3B,C). Given that the DRG and spinal dorsal horn (SDH) are key regions involved in nociception and the integration of pain signals, these regions were then excised (Figure 3D), and IVIS imaging confirmed mFLuc expression in both organs (Figure 3E,F).^[^ ^13^ ^]^ Higher bioluminescence signals were observed in the excised spinal cord and DRG tissues in the P6CIT/mFLuc group compared to the MC3/mFLuc group.

**Figure 4:**
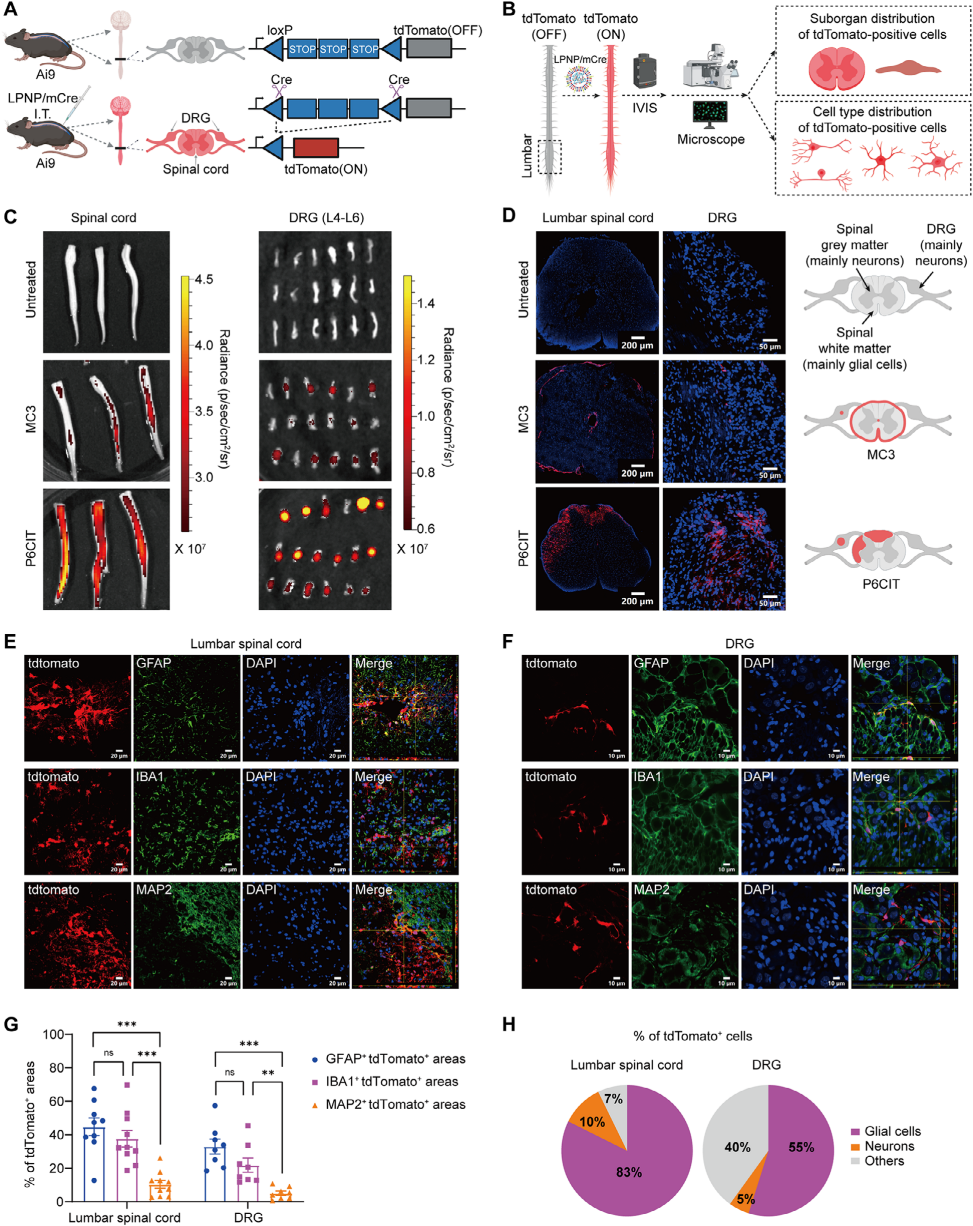
Transfection efficiency and cell type specificity in the spinal cord and DRG of Ai9 mice following the intrathecal injection of mCre‐loaded LPNPs. A) Schematic of the intrathecal delivery of mCre‐loaded LPNPs in Ai9 mice and Cre‐mediated recombination of the tdTomato gene. B) Procedure for assessing tdTomato expression. C) IVIS images of the spinal cord (left) and DRG (right) from Ai9 mice treated with MC3/mCre or P6CIT/mCre. *n* = 3. D) Fluorescence images and schematic of the tdTomato‐positive areas in the lumbar spinal cord and DRG of Ai9 mice treated with mCre‐loaded LPNPs. Scale bars = 200 µm (left) and 50 µm (right). E,F) Representative immunofluorescence images showing the colocalization of GFAP (green), IBA1(green), and MAP2 (green) with tdTomato (red) in E) the lumbar spinal cord and F) DRG of Ai9 mice treated with P6CIT/mCre. E) Scale bar = 20 µm and F) Scale bar = 10 µm. G) Percentages of GFAP+ tdTomato+ cells, IBA1+ tdTomato+ cells, and MAP2+ tdTomato+ cells among total tdTomato+ cells in the lumbar spinal cord and DRG of Ai9 mice treated with P6CIT/mCre. *n* = 8–10 random fields from 3 mice. H) Cell type distribution of tdTomato‐positive cells in the lumbar spinal cord and DRG of Ai9 mice treated with P6CIT/mCre. Statistical analysis was performed using one‐way ANOVA in (G). The data are expressed as the mean ± SEM; *p < 0.05, **p < 0.01, ***p < 0.001; ns, nonsignificant.

Ai9 mice carry a fluorescent tdTomato gene that is controlled by loxP‐flanked STOP cassettes. Upon activation by Cre recombinase, the tdTomato gene is expressed, resulting in fluorescence in Cre mRNA‐expressing cells (**Figure** **4**A). Ai9 mice received intrathecal injections of Cre mRNA‐loaded nanoparticles every other day, for a total of two injections. To assess tdTomato expression, spinal cord and DRG tissues were collected one week after the initial injection for analysis using IVIS and microscopy (Figure 4B). IVIS imaging revealed significantly higher tdTomato fluorescence in the P6CIT/mCre group compared to the control group and the MC3/mCre group in both the spinal cord and DRG tissues (Figure 4C and Figure S2, Supporting Information). Confocal microscopy images further confirmed that the P6CIT/mCre group exhibited the highest number of tdTomato‐positive cells in the lumbar spinal cord and DRG, in comparison to the MC3/mCre and control groups (Figure 4D). In the P6CIT/mCre group, tdTomato‐positive cells were abundant in the white matter of the spinal cord, whereas in the MC3/mCre group, these cells were concentrated at the edge of the spinal cord (Figure 4D). The deeper distribution of mRNA expression further suggested that the P6CIT lipopolymer has higher penetration of pia mater and subpial tissues compared to the MC3 ionizable lipid (Figure 4D).

To determine the cell types that were transfected by our P6CIT LPNPs, we conducted immunofluorescence staining of spinal cord and DRG tissues after Cre mRNA delivery. One‐week after the intrathecal injection of P6CIT/mCre into Ai9 mice, the lumbar spinal cord and DRG tissues were fixed, cryosectioned, and stained. In the lumbar spinal cord, GFAP, IBA1, and MAP2 antibodies were used to label astrocytes, microglia, and neurons, respectively. In the DRG, GFAP, IBA1, and MAP2 antibodies were used to label GFAP‐expressing glial cells, IBA1‐positive macrophage‐like cells, and neurons, respectively.^[^ ^3d,e^ ^]^ In both the lumbar spinal cord and DRG, GFAP, IBA1, and MAP2 were all colocalized with tdTomato (Figure 4E,F). The percentages of GFAP+ tdTomato+ areas (44.7% ± 5.3%) and IBA1+ tdTomato+ areas (37.7% ± 5.0%) in the tdTomato+ areas were significantly higher than the MAP2+ tdTomato+ areas (10.4% ± 2.4%) in the lumbar spinal cord (Figure 4G). The percentages of GFAP+ tdTomato+ areas (33.0% ± 4.5%) and IBA1+ tdTomato+ areas (21.9% ± 4.2%) in the tdTomato+ areas were also significantly higher than the MAP2+ tdTomato+ areas (5.1% ± 1.3%) in the DRG (Figure 4G). These findings suggest that P6CIT/mCre was primarily absorbed and expressed in gliocytes rather than in neurons (Figure 4H). The co‐localization of tdTomato with GFAP did not show a significant difference when compared to its co‐localization with IBA1 (Figure 4G).

### 2.3 P6CIT LPNPs Successfully Delivered mIL‐10 into the Spinal Cord and DRG

**Figure 5:**
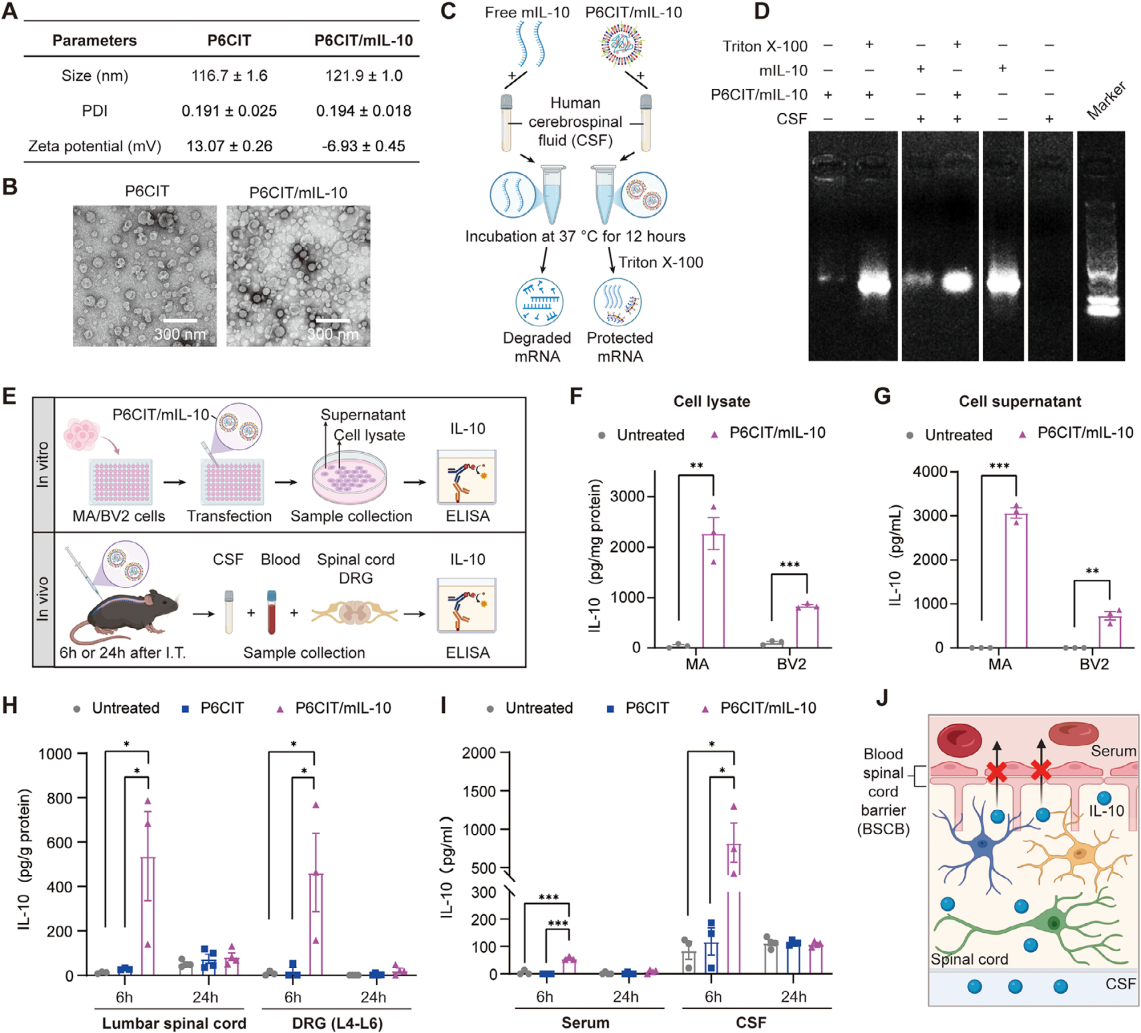
Characterization and gene expression of P6CIT/mIL‐10. A) Average particle size, PDI and zeta potential measurements of P6CIT and P6CIT/mIL‐10. *n* = 3. B) TEM images of P6CIT and P6CIT/mIL‐10. Scale bar = 300 nm. C) Procedure for assessing the stability of P6CIT/mIL‐10 in human CSF. D) Stability comparison of free IL‐10 mRNA and mIL‐10 encapsulated in P6CIT LPNPs after incubation with human CSF for 12 h. E) Schematic of mIL‐10 expression validation in vitro and in vivo. ELISA analysis of F) the intracellular IL‐10 levels and G) supernatant IL‐10 levels in MA cells and BV2 cells transfected with P6CIT/mIL‐10. *n* = 3. ELISA analysis of IL‐10 levels in H) the lumbar spinal cord and DRG tissues, and I) in the serum and CSF of C57BL/6J mice at 6 hours or 24 h after the intrathecal injection of P6CIT LPNPs or P6CIT/mIL‐10. *n* = 3–4. J) Schematic of the blood‐spinal cord barrier (BSCB). Statistical analysis was performed via two‐tailed unpaired *t*‐tests for (F,G), and one‐way ANOVA for (H,I). The data are expressed as the mean ± SEM; *p < 0.05, **p < 0.01, ***p < 0.001; ns, nonsignificant.

After confirmation of the delivery efficiency of the P6CIT LPNPs, mIL‐10 was synthesized and encapsulated into P6CIT LPNPs to form P6CIT/mIL‐10, after which a comprehensive evaluation was performed, including characterization, in vitro cellular assays, and in vivo animal experiments.^[^ ^14^ ^]^ The mIL‐10 was observed to be greater than 500 bases in length (Figure S3, Supporting Information). The hydrodynamic size, PDI, and zeta potential of P6CIT LPNPs were 116.7 ± 1.6 nm, 0.191 ± 0.025, and 13.07 ± 0.26 mV, respectively (**Figure** **5**A). After encapsulation of IL‐10 mRNA, the hydrodynamic size increased to 121.9 ± 1.0 nm and the zeta potential decreased to −6.93 ± 0.45 mV, respectively (Figure 5A). TEM images revealed that both P6CIT LPNPs and P6CIT/mIL‐10 had uniform and spherical structures (Figure 5B).

We then evaluated the stability of P6CIT/mIL‐10 in human CSF (Figure 5C). After incubation with human CSF at 37 °C for 12 h, P6CIT/mIL‐10 demonstrated excellent stability, with the size and PDI remaining at approximately 100 nm and 0.2, respectively (Table S3, Supporting Information). Electrophoresis results showed that P6CIT LPNPs efficiently encapsulated mIL‐10, as evidenced by the nearly undetectable band in the lane for P6CIT/mIL‐10 (Figure 5D). A positive band appeared when mIL‐10 was extracted from P6CIT/mIL‐10 by 1% Triton X‐100 (Figure 5D). Human CSF did not contain any free mIL‐10 (Figure 5D). Moreover, free mIL‐10 was rapidly degraded within twelve hours of incubation with human CSF at 37 °C, while mIL‐10 encapsulated within P6CIT LPNPs maintained excellent stability (Figure 5D). The mIL‐10 encapsulated within P6CIT LPNPs exhibited a gradual decline in stability as the duration of incubation with human CSF increased (Figure S4, Supporting Information).

The translational efficacy of IL‐10 mRNA delivered by P6CIT LPNPs was evaluated using ELISA both in vitro and in vivo (Figure 5E). In both MA and MV2 cells, treatment with P6CIT/mIL‐10 markedly increased the IL‐10 expression levels within the cells (Figure 5F) and in the cellular supernatant (Figure 5G). Moreover, IL‐10 expression levels in the lumbar spinal cord, DRG, serum, and CSF of C57BL/6J mice were significantly elevated six hours following a single intrathecal injection of P6CIT/mIL‐10, but returned to baseline levels after 24 hours (Figure 5H,I). Notably, intrathecal injection predominantly increased IL‐10 expression in the CSF but not in the serum (Figure 5I). This finding suggested that this administration route of administration may prevent P6CIT/mIL‐10 and IL‐10 from crossing the blood‐spinal cord barrier (BSCB) (Figure 5J), thereby potentially reducing side effects on organs located outside of the CNS. In contrast, P6CIT LPNPs alone did not induce significant changes in IL‐10 expression in the spinal cord, DRG, serum, or CSF of mice following a single intrathecal injection (Figure 5H,I).

### 2.4 Intrathecal Administration of P6CIT/mIL‐10 Ameliorates PIPN by Inhibiting Glial Cell Activation and Presynaptic NMDAR Hyperactivity

**Figure 6:**
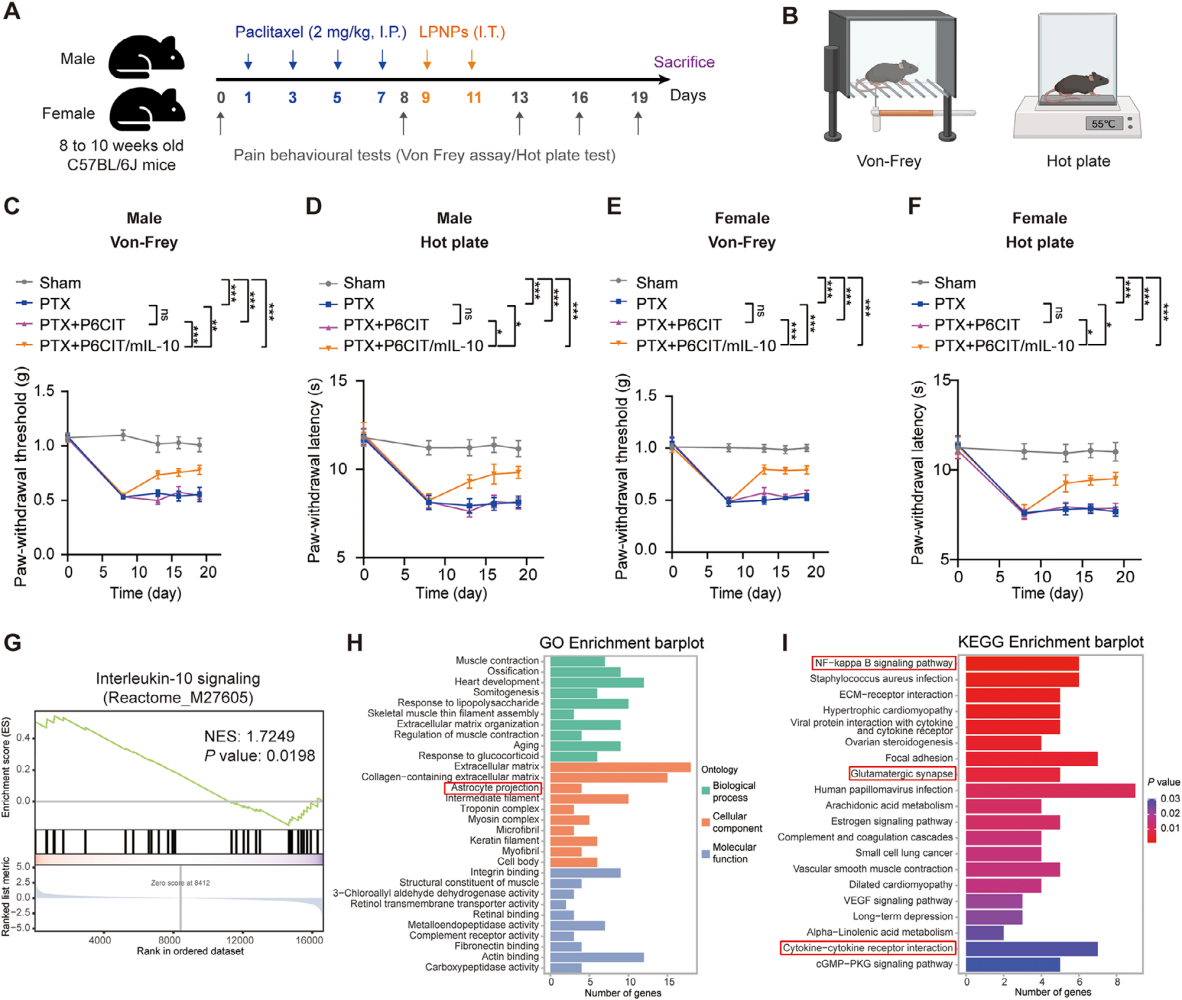
Therapeutic effects of P6CIT/mIL‐10 on pain relief in PIPN mice and high‐throughput RNA sequencing results. A) Schematic illustration of paclitaxel treatment, LPNP treatment, behavioral assessment, and tissue collection in C57BL/6J mice. B) Schematic of the Von‐Frey test and the hot plate test. Measurement of mechanical allodynia in C) male mice and E) female mice following paclitaxel and LPNPs treatments. *n* = 10 mice per group. Measurements of thermal hyperalgesia in D) male mice and F) female mice following paclitaxel and LPNPs treatments. *n* = 10 mice per group. G) GSEA of the IL‐10 signaling pathway in the DRG of mice treated with PTX + P6CIT/mIL‐10 compared to those treated with PTX alone. H) GO enrichment of significantly differentially expressed genes in the DRG of mice treated with PTX + P6CIT/mIL‐10 compared to those treated with PTX alone. I) Top 20 enriched KEGG pathways of significantly differentially expressed genes in the DRG of mice treated with PTX + P6CIT/mIL‐10 compared to those treated with PTX alone. Statistical analysis was performed via two‐way ANOVA for (C–F). The data are expressed as the mean ± SEM; *p < 0.05, **p < 0.01, ***p < 0.001; ns, nonsignificant. Two‐way ANOVA followed by Tukey's post hoc test.

We then evaluated the therapeutic efficacy of P6CIT/mIL‐10 in a C57BL/6J mouse PIPN model (**Figure** **6**A). Considering the sex‐specific sensitivity of PIPN, we administered PTX and P6CIT/mIL‐10 to both male and female mice.^[^ ^15^ ^]^ Mechanical allodynia was assessed using the Von‐Frey test, and thermal hyperalgesia was evaluated with the hot plate test (Figure 6B). Following PTX treatment, both male and female mice exhibited persistent pain behaviors, which were characterized by significant and prolonged reductions in the paw‐withdrawal threshold and paw‐withdrawal latency (Figure 6C–F). Two intrathecal injections of P6CIT LPNPs did not significantly affect paclitaxel‐induced nociceptive behaviors (Figure 6C–F). In contrast, treatment with P6CIT/mIL‐10 significantly improved the paclitaxel‐induced decreases in both the paw withdrawal threshold and latency in both male and female mice, indicating a reduction in mechanical and thermal allodynia (Figure 6C–F).

To explore the mechanisms underlying the therapeutic effects of P6CIT/mIL‐10 on PIPN, high‐throughput RNA sequencing (RNAseq) was conducted on DRG tissues from the PTX group and the PTX+P6CIT/mIL‐10 group. Approximately 50 million reads per sample were generated. Differential expression analysis between the PTX+P6CIT/mIL‐10 group and the PTX group identified 231 significantly differentially expressed genes (DEGs). Of these, 54 genes were upregulated in the PTX+P6CIT/mIL‐10 group, and 177 genes were downregulated (Figure S5, Supporting Information). GSEA demonstrated significant upregulation of IL‐10 signaling in the DRG following the intrathecal injection of P6CIT/mIL‐10 in the presence of PTX (Figure 6G). GO analysis, focusing on cellular components (CC), revealed notable enrichment of DEGs in targets associated with astrocyte projection (Figure 6H). KEGG analysis revealed that DEGs were enriched in pathways associated with “NF‐𝜅B signaling pathway”, “Glutamatergic synapse”, and “Cytokine‐cytokine receptor interaction” (Figure 6I).

**Figure 7:**
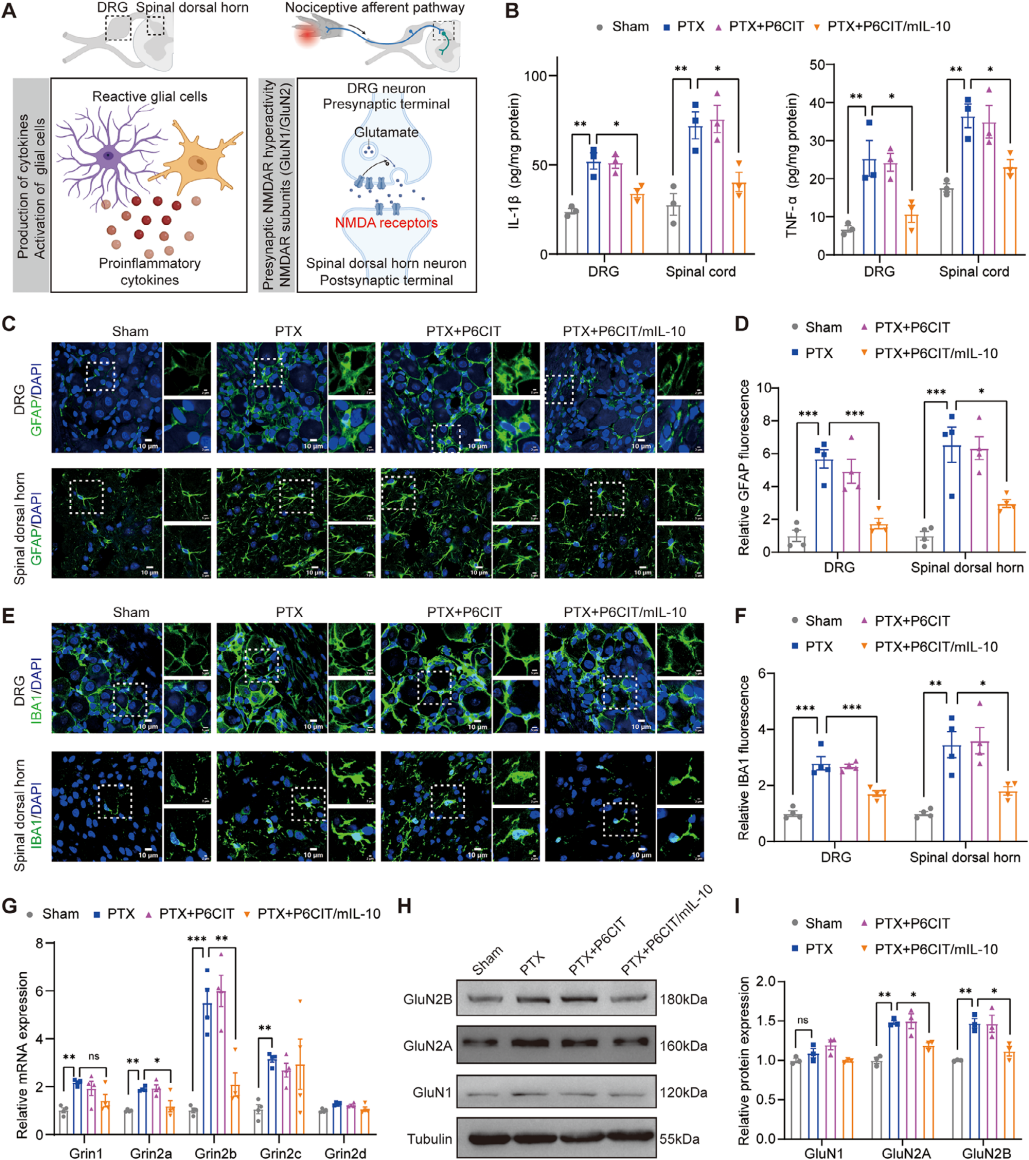
Effects of P6CIT/mIL‐10 on the alleviation of proinflammatory cytokine production, glial cell activation, and presynaptic NMDAR hyperactivity in PIPN mice. A) Schematic of the underlying mechanisms of P6CIT/mIL‐10 for pain relief in PIPN mice. B) ELISA analysis of IL‐1β and TNFα levels in the lumbar spinal cord and DRG of mice following different treatments. *n* = 3. C) Representative immunofluorescence images and D) quantitative analysis of GFAP (green) in the SDH and DRG of mice following different treatments. *n* = 4. Scale bar = 10 µm. E) Representative immunofluorescence images and F) quantitative analysis of IBA1 (green) in the SDH and DRG of mice following different treatments. *n* = 4. Scale bar = 10 µm. G) mRNA expression levels of Grin1, Grin2a, Grin2b, Grin2c and Grin2d in the DRG of mice following different treatments. *n* = 4. H) Representative immunoblots and I) quantitative analysis of GluN1, GluN2A, and GluN2B in the DRG of mice following different treatments. *n* = 3. Statistical analysis was performed using one‐way ANOVA in (B, D, F, G, and I). The data are expressed as the mean ± SEM; *p < 0.05, **p < 0.01, ***p < 0.001; ns, nonsignificant.

To further confirm the RNA‐seq results, the production of proinflammatory cytokines, glial cell activation, and the presynaptic NMDAR activity were evaluated, as shown in **Figure** **7**. PTX significantly increased IL‐1β and TNFα production in the lumbar spinal cord and DRG, and these effects were reversed by P6CIT/mIL‐10 treatment (Figure 7B). The administration of PTX or P6CIT/mIL‐10 did not significantly alter the levels of IL‐6 or TGF‐β in the lumbar spinal cord or DRG (Figure S6, Supporting Information). Additionally, PTX administration increased the expression levels of GFAP and IBA1 (Figure 7C–F) in the SDH, indicating the activation of astrocytes and microglia. In the DRG, PTX increased GFAP and IBA1 expression, suggesting the activation of GFAP‐expressing glial cells and the infiltration of IBA1‐positive microphage‐like cells (Figure 7C–F). P6CIT LPNPs without mIL‐10 treatment did not significantly alter GFAP or IBA1 levels in the presence of PTX (Figure 7C–F). In contrast, P6CIT/mIL‐10 treatment significantly reduced the overexpression of GFAP (Figure 7C,D) and IBA1 (Figure 7E,F) induced by PTX in both the SDH and DRG. Besides, PTX caused microglial de‐ramification and astrocyte hypertrophy in the SDH, representing reactive morphological changes, which were reversed by P6CIT/mIL‐10 treatment (Figure 7C,E). Furthermore, in the DRG, PTX increased the mRNA expression of NMDAR subunits, including Grin1, Grin2a, Grin2b and Grin2c, indicating an increase in presynaptic glutamate release to neurons in the SDH (Figure 7G). P6CIT/mIL‐10 treatment reduced the elevated mRNA levels of Grin2a and Grin2b induced by PTX, while the mRNA levels of Grin1 and Grin2c remained unchanged (Figure 7G). PTX also raised the protein levels of GluN2A and GluN2B in the DRG; however, these levels decreased with P6CIT/mIL‐10 treatment (Figure 7H,I). Protein levels of GluN1 did not show significant difference following PTX or P6CIT/mIL‐10 treatment (Figure 7H,I).

### 2.5 Biosafety Evaluation of P6CIT LPNPs

The safety of P6CIT LPNPs was evaluated both in vitro and in vivo. No significant cytotoxicity was observed in P6CIT LPNP‐treated MA or BV2 cells compared with the control group (Figure S7, Supporting Information). Additionally, there were no increases in the levels of GFAP, IBA1, or caspase 3 in the spinal cord and DRG of C57BL/6J mice at one day, one week, or one month after intrathecal injection of P6CIT LPNPs (Figure S8A, Supporting Information), indicating that the intrathecal injection of P6CIT LPNPs was not able to induce glial cell activation and cell apoptosis. H&E staining showed no signs of injury in major organs (heart, liver, spleen, lung, and kidney) of mice at one day, one week, or one month after the intrathecal injection of P6CIT LPNPs (Figure S8B, Supporting Information). Taken together, P6CIT LPNPs were selected for further treatment studies.

## 3 Discussion

The high incidence of PIPN observed in cancer patients undergoing PTX treatment is coupled with limited effective management options.^[^ ^2a^ ^]^ Recently, mRNA therapeutics have been explored for treating neurological disorders. Intracerebral injections of mRNA‐loaded LPNPs have been shown to transfect approximately 50% of cells within the striatum and hippocampus.^[^ ^16^ ^]^ However, intracerebral delivery causes significant damage to the brain, limiting its clinical application. As an alternative, the intrathecal administration of therapeutic agents offers a less invasive approach to modulate cells in the brain and spinal cord and is commonly used in clinical practice. However, the intrathecal delivery of mRNA is also limited by the biological barriers of the pia mater. In our study, we intrathecally delivered IL‐10 mRNA encapsulated in P6CIT LPNPs to treat PIPN.

Nanoparticles that are capable of consistently crossing the BSCB have been developed for the treatment of spinal cord diseases.^[^ ^17^ ^]^ However, the ability of nanoparticles to cross the CSF‐brain/spinal cord barrier remains largely unexplored. Previous studies have shown that larger molecules exhibit lower permeability across the CSF‐brain barrier compared to smaller molecules.^[^ ^9b^ ^]^ Our findings suggest that the enhanced penetration capability of P6CIT LPNPs, compared to MC3 LNPs, may be attributed to their smaller particle size. Additionally, the interstitial fluid surrounding the brain and spinal cord carries a negative charge,^[^ ^18^ ^]^ which could further explain the diminished penetration capability of MC3/mCre, as it possesses a more negative zeta potential compared to P6CIT/mCre. Our study also demonstrates successful transfection and gene expression of mRNA‐loaded P6CIT LPNPs in the spinal cord and DRG of mice by intrathecal injection, which was superior to the effects of MC3 LNPs. The intrinsic physicochemical properties of lipids, including headgroup chemistry, hydrophobic tail chain length, degree of branching, and saturation level, directly modulate their transfection efficiency.^[^ ^19^ ^]^ P6CIT features shorter hydrophobic chains and exhibits stronger lipophilicity compared to MC3, which enhances its cellular internalization and endosomal escape capabilities, thereby leading to higher delivery efficiency. Moreover, P6CIT LPNPs primarily transfected astrocytes and microglia in the spinal cord, with limited neuronal transfection. In contrast, MC3 LNPs, administered intracerebrally, predominantly transfect astrocytes and neurons in the striatum and hippocampus.^[^ ^16^ ^]^

The intrathecal delivery of IL‐10 mRNA by our P6CIT LPNPs also exhibited restricted expression of IL‐10, which benefited the local therapeutic outcomes and reduced systematic side effects. The BBB and BSCB in the CNS, which are composed of endothelial cells with specialized tight junctions, protect neural tissue by limiting the passage of substances across these barriers.^[^ ^29^ ^]^ Intrathecal delivery of LPNPs allows encapsulated mRNA to be primarily expressed within CNS tissues, enabling effective mRNA‐based therapies with minimal side effects on non‐CNS organs in treating neurological disorders. The slight increase in serum IL‐10 observed in mice following the intrathecal injection of P6CIT/mIL‐10 may be attributed to leakage of fluid during the withdrawal of the micro‐syringe at the end of the injection.

The mechanism for IL‐10 mRNA therapy was also evaluated in our study. Paclitaxel induces microglial and astrocytic activation, which promotes the release of proinflammatory cytokines, leading to neuronal damage and glutamatergic neuronal hyperexcitability.^[^ ^2^, ^21^ ^]^ Complementary therapies using IL‐10 have been shown to reduce paclitaxel‐induced immune responses,^[^ ^5a,b^ ^]^ which aligns with our current findings. IL‐10 has also been found to suppress abnormal paclitaxel‐induced spontaneous discharges and stimulate the Nrf2‐HO1 pathway in DRG neurons.^[^ ^5^, ^22^ ^]^ Intrathecal injection of P6CIT/mIL‐10 alleviated alleviates presynaptic NMDAR hyperactivity, which may be partly attributed to decreased glial cell activation and decreased production of proinflammatory cytokines. Previous research has demonstrated that the IL‐10 receptor (IL‐10R) is expressed on the membrane of DRG sensory neurons.^[^ ^23^ ^]^ These findings suggest that exogenous IL‐10 may also bind to IL‐10R on the membrane of DRG neurons and then elicit neuroprotective effects in PIPN mice treated with P6CIT/mIL‐10. Further research is required to investigate the mechanism of IL‐10 in DRG neurons.

Although we have demonstrated the effective transfection of LPNPs in the spinal cord and DRG, as well as the therapeutic effects of engineered P6CIT/mIL‐10 in PIPN mice, several limitations should be noted. First, intrathecal injection allows LPNPs to circulate freely in the CSF, which flow throughout the CNS. This distribution could potentially lead to unintended side effects in brain tissues. Additionally, presynaptic NMDAR hyperactivity in DRG neurons amplifies nociceptive input to SDH neurons.^[^ ^4a^ ^]^ Unfortunately, due to technical constraints, we were unable to use whole‐cell patch‐clamp techniques to record excitatory postsynaptic currents in SDH neurons.

## 4 Conclusions 

In summary, we developed a pia mater‐penetrable P6CIT LPNP for gliocyte‐targeted delivery of IL‐10 mRNA to treat PIPN. Our self‐developed P6CIT LPNPs exhibited superior transfection efficacy and CSF‐spinal cord barrier penetration compared to commercial MC3 LNPs both in vitro and in vivo. Intrathecal injection of engineered P6CIT/mIL‐10 showed significant therapeutic efficacy in PIPN mice. These findings provide valuable insights into advancing mRNA therapeutics for the treatment of spinal cord‐ and DRG‐related diseases.

## 5 Experimental Section

### Materials

1,2‐Distearoyl‐sn‐glycero‐3‐phosphocholine (DSPC), 1,2‐dimyristoyl‐rac‐glycero‐3‐methoxy polyethylene glycol‐2000 (DMG‐PEG 2000), Dlin‐MC3‐DMA (MC3), and D‐Luciferin sodium were purchased from Bide (Shanghai, China). The linearization kit and IVT kit were purchased from Changchun Golden Transfer Science and Technology Co., Ltd. (Changchun, China). Cholesterol and DAPI were purchased from Solarbio (Beijing, China). The Firefly Luciferase mRNA (mFLuc), EGFP mRNA (mEGFP) and Cre mRNA (mCre) were purchased from MagicRNA (Shenzhen, China). A luciferase reporter gene assay kit, GelRed, SYBR GOLD, and cell counting kit (CCK8) were purchased from Yeasen (Shanghai, China). TRIzol reagent, the PrimeScript RT Reagent Kit, and SYBR Green reaction mix were purchased from Vazyme (Nanjing, China). Paclitaxel was purchased from MedChemExpress (Monmouth Junction, USA). Lipofectamine 3000 (Lipo3000) was purchased from ThermoFisher (Waltham, USA). RIPA lysis buffer, protease inhibitors, and phosphatase inhibitors were purchased from Beyotime (Shanghai, China). Transwell chambers and Matrigel were purchased from Corning (Corning, USA). The DNA ladder was purchased from CWBIO (Beijing, China), and the ssRNA ladder was purchased from NEB (Ipswich, USA). Human CSF was obtained from Sun Yat‐Sen Memorial Hospital with patient consent and ethical approval.

### Plasmid Extraction and In Vitro Transcription (IVT) of mRNA

The coding sequence of mouse IL‐10 mRNA (mIL‐10) was referred to as NCBI (gene ID 22262) (Table S1, Supporting Information). In vitro transcription (IVT) was performed using the Standard mRNA Synthesis kit (E2060s, NEB). The linearization of the plasmid containing the IL‐10 sequence was carried out using BspQ1 digestion, and the linearized plasmid was taken as an IVT template. IVT was carried out as indicated by the manufacturer's protocol with 3‐OH AG (cap1). The VAHTS mRNA capture beads were used to purify the final transcribed mRNAs, and the final mRNA concentration was determined using Nanodrop.

### Synthesis and Characterization of LPNPs

The P6CIT lipopolymer was synthesized by the reaction of branched polyethyleneimine (PEI) (*M*W = 600) between 3,7‐dimethyl‐6‐octen‐1‐yl acrylate at a molar ratio of 1:28 at 75 °C for 3 d. The LPNP was formulated with cholesterol, DSPC, and DMG‐PEG2000 at the weight ratio of 16:12:8:4. The hydrodynamic size, polydispersity index (PDI), and zeta potential of LPNPs were determined using dynamic light scattering (DLS, Malvern, UK). The morphology of LPNPs was determined using transmission electron microscopy (TEM).

### Cell Culture

The BV2 mouse microglial cell line and the MA mouse astrocytic cell line were purchased from American Type Culture Collection (ATCC). The cells were cultured in Dulbecco's modified Eagle's medium (DMEM, Gibco, USA) supplemented with 10% fetal bovine serum (FBS, Excellbio, China) and 1% penicillin and streptomycin (Gibco, USA) at 37 °C with 5% CO2.

### Cellular Uptake Activity

BV2 cells and MA cells were seeded and cultured on 24‐well plates overnight. Free DID (0.8 µg mL^−1^) or LPNPs encapsulating 2% DID were incubated with cells at 37 °C for 1 h. Next, the cells were digested, washed with PBS, and analyzed using a CytoFLEX flow cytometer (Beckman, USA).

### In Vitro Transfection of LPNPs

Cells were seeded overnight and then transfected with mRNA‐loaded LPNPs at 1 µg mRNA per well. After 24 hours, the cells were collected for analysis: EGFP fluorescence was examined using an inverted fluorescence microscope (Olympus IX73), luciferase activity was measured via luciferase reporter gene assay kits with a BioTek Synergy H1 Multi‐Mode Microplate Reader (Agilent), and the IL‐10 concentration was assessed via ELISA assays.

### Transwell Assays

Primary meningeal cells were isolated from C57BL/6J mouse pups on postnatal day 0–1, as previously described.^[^ ^23^ ^]^ The leptomeninges were carefully dissected from the cerebral hemispheres under a microscope and digested in 0.125% trypsin for 20 min at 37 °C. The cells were then centrifuged at 1000 × *g* for 5 min and resuspended in cell culture medium. Cells were maintained in DMEM supplemented with 10% FBS and 1% penicillin and streptomycin.

Transwell assays were performed using Transwell chambers, primary meningeal cells, and Matrigel. To evaluate the penetration of LPNPs into the pia mater, primary meningeal cells were seeded in the upper chamber of the Transwell system with complete medium. LPNPs containing DID were added to the upper chamber and incubated for 2 hours. After incubation, the mean fluorescence intensity (MFI) of DID in the lower chamber was measured using an in vivo imaging system (IVIS) or a Microplate Reader. Additionally, LPNPs containing EGFP mRNA were added to the upper chamber, and after 24 h of incubation, the fluorescence of EGFP in the MA cells inoculated in the lower chamber was assessed using a confocal laser scanning microscope (Olympus FV3000).

To evaluate the penetration of LPNPs into subpial tissues, the bottom of the upper chamber of the Transwell system was coated with Matrigel, and LPNPs containing DID were added to the upper chamber. The MFI of DID in the lower chamber was measured at predefined time points using a Microplate Reader. Additionally, after 24 h of incubation, the MFI of DID in the MA cells inoculated in the lower chamber was assessed using a CytoFLEX flow cytometer.

### mRNA LPNPs Stability Assessment

Agarose gel electrophoresis was used to evaluate the protective capability of P6CIT LPNPs in preventing mIL‐10 degradation in human CSF. Briefly, free mIL‐10 or P6CIT/mIL‐10 was incubated with CSF at 37 °C. P6CIT/mIL‐10 was incubated with 1% Triton X‐100 to extract the encapsulated mRNA. At 12 hours, the hydrodynamic sizes of LPNPs were determined by DLS. Afterwards, mIL‐10 was extracted from P6CIT/mIL‐10, electrophoresed on a 1.5% agarose gel stained with GelRed, and visualized using the ChemiDoc system (Bio‐Rad, USA). A DNA marker was used to roughly estimate the IL‐10 mRNA's position.

### Animals

Eight‐ to ten‐week‐old female and male C57BL/6J mice and ICR mice were purchased from Zhuhai BesTest Bio‐Tech Co., Ltd. Ai9 mice were obtained from The Jackson Laboratory (Stock number: 007909). The use of animals was approved by the Animal Care and Use Committees of the Laboratory Animal Research Center at Sun Yat‐Sen Memorial Hospital, Sun Yat‐Sen University (No. AP20240032, No. AP20240156).

### Establishment of the PIPN Model and Treatment by Intrathecal Injection

A mouse model of PIPN was established following previously described methods.^[^ ^24^ ^]^ In brief, paclitaxel was prepared as a stock solution at a concentration of 6 mg mL^−1^ by diluting it in a mixture of 50% Cremophor EL and 50% absolute ethanol. The stock solution was further diluted with 0.9% saline to achieve a concentration of 1 mg mL^−1^ and was intraperitoneally administered to the mice at a dose of 2 mg kg^−1^ on Days 1, 3, 5, and 7. Mice in the sham group received injections of an equivalent volume of saline.

Mice were anesthetized with sodium pentobarbital (50 mg kg^−1^) and positioned horizontally. A 10 µL Hamilton micro‐syringe was carefully inserted into the subarachnoid space between the L5 and L6 vertebrae. A slight wag or flick of the tail reflected a successful injection. A volume of 5 µL of LPNPs was used for each intrathecal injection.

### Behavior Tests: Von Frey Assay

Mechanical allodynia was assessed using Von Frey tests (KEW Basis, China).^[^ ^25^ ^]^ Briefly, mice were put into a transparent box with a grid floor and allowed to acclimate for 15 min. A calibrated filament was gently applied to the plantar surface of the right hind paw, and pressure was gradually increased until a paw withdrawal response (such as sudden claw retraction, shaking or licking) was observed. The paw withdrawal threshold was quantitatively measured using an electronic Von Frey esthesiometer. Each mouse underwent five trials, and the average of these five measurements was recorded as the final paw withdrawal threshold.

### Hot Plate Test

Thermal hyperalgesia was measured by a hot plate test using a thermal plantar analgesia apparatus (KEW Basis, China).^[^ ^26^ ^]^ Briefly, mice were placed on a hot plate heated to 55 °C, and a timer was started at the moment of exposure. The timer was stopped when the mice exhibited a response, such as lifting their paws, licking, or jumping. To prevent tissue injury, a maximum cutoff time of 20 s was set. Each mouse underwent two trials, with at least a 5 min interval occurring between the tests, and the paw withdrawal latency was obtained by averaging the two measurements.

### Collection of Mouse CSF, Serum, Spinal Cord Tissue, and DRG Tissue

Mouse CSF was collected following a previously described method.^[^ ^27^ ^]^ Briefly, mice were anesthetized and secured in a stereotactic instrument. The cisterna magna was exposed and punctured with a 1 mL insulin syringe, thus allowing for approximately 10 µL of CSF to be withdrawn. Blood was collected from the retro‐orbital plexuses by carefully removing the eyeballs, followed by storage at room temperature for 1 h. The samples were then centrifuged at 3000 × *g* for 15 min to obtain serum (clear supernatant). Finally, the mice were intracardially perfused with 50 mL of 0.9% saline. Following perfusion, a 5 mL syringe filled with 0.9% saline was used to gently flush the spinal cord out of the spine. The bilateral lumbar DRG (L4–L6) tissues were then carefully dissected from the spine under a stereomicroscope.

### RNA‐Seq

DRG tissues from three mice were pooled to form a single sample. Two samples from the PTX+P6CIT/mIL‐10 group and one sample from the PTX group were used for whole transcriptome sequencing. A total of 1 µg of RNA per sample was used as input for RNA sample preparation. Sequencing libraries were created using NEBNext Ultra RNA Library Prep Kit for Illumina (NEB, USA). Index‐coded samples were clustered on a cBot Cluster Generation System with the TruSeq PE Cluster Kit v3‐cBot‐HS (Illumina). Following cluster generation, libraries were sequenced on an Illumina NovaSeq platform, and 150 bp paired‐end reads were generated. Subsequent analyses were performed using high‐quality clean data. The FPKM for each gene was calculated based on gene length and the number of reads mapped to it. Differentially expressed genes were analyzed for Gene Ontology (GO) enrichment, Kyoto Encyclopedia of Genes and Genomes (KEGG) pathway enrichment, and Gene Set Enrichment Analysis (GSEA) using established methodologies.^[^ ^28^ ^]^

### Statistical Analysis

All of the statistical analyses were conducted using GraphPad Prism software. Data are expressed as means ± standard errors of the means (SEMs). To assess differences between two groups, a two‐tailed Student's t‐test was used. For comparisons across multiple groups, one‐way or two‐way analysis of variance (ANOVA) followed by Tukey's post hoc test was applied. A *p*‐value of less than 0.05 was considered to be statistically significant (*p < 0.05, **p < 0.01, and ***p < 0.001).

## Conflict of Interest

The authors declare no conflict of interest.

## Author Contributions

X.L., L.W., and X.L. contributed equally to this work. X.L., J.C., and Y.P. were involved in the conceptualization of the study. X.L., L.W., X.L., L.Z., Y.T., H.W., H.L., C.L., and H.W. performed the experiments and analyzed the data. J.C., X.L., and Y.P. supervised the project. X.L., L.W., J.C., and Y.P. prepared the figures, and edited the manuscript. J.C. and Y.P. acquired funding for the studies. All authors contributed to the article and approved the submission of the manuscript.

## Consent to Participate

The patient consent and ethical approval were obtained from the Institutional Ethics Committee of Sun Yat‐Sen Memorial Hospital, Sun Yat‐Sen University.

## Supporting information



Supporting Information

## Data Availability

The datasets used and/or analyzed during the current study are available from the corresponding author upon reasonable request.
