# Supplementary material for: Pia Mater‐Penetrable Lipopolymer Nanoparticles for Gliocyte‐Targeted IL‐10 mRNA Therapy Alleviate Paclitaxel‐Induced Peripheral Neuropathy
Source: Adv Sci (Weinh). 2025 May 13;12(23):2500362. doi: 10.1002/advs.202500362 (PMC12199344; doi:10.1002/advs.202500362)
Supplement: Supplementary file 1 — Supporting Information [file ADVS-12-2500362-s001.docx]

**Pia mater-penetrable lipopolymer nanoparticles for gliocytes-targeted IL-10 mRNA therapy alleviate paclitaxel-induced peripheral neuropathy**

Xinrou Lin^1,2,6†^, Lingyu Wei^2,3†^, Xiangpen Li^1,4†^, Ling Zeng^2,3^, Yingsen Tang^2,3^, Hongjin Wang^5^, Hengjian Lu^1^, Chenguang Li^1^, Hongxuan Wang^1^, Jinjin Chen^1,2,3*^, Ying Peng^1,2,3*^

1, Department of Neurology, Sun Yat-Sen Memorial Hospital, Sun Yat-Sen University, Guangzhou, China

2, Nanhai Translational Innovation Center of Precision Immunology, Sun Yat-Sen Memorial Hospital, Foshan 528200, China

3, Guangdong Provincial Key Laboratory of Malignant Tumor Epigenetics and Gene Regulation, Sun Yat-Sen Memorial Hospital, Sun Yat-Sen University, Guangzhou, China

4, Shenshan Medical Center, Sun Yat-Sen Memorial Hospital, Sun Yat-Sen University, Shanwei, China

5, Department of Urology, Sun Yat-Sen Memorial Hospital, Sun Yat-Sen University, Guangzhou, China

6, Department of Anesthesiology, Sun Yat-Sen Memorial Hospital, Sun Yat-Sen University, Guangzhou, China

† These authors contributed equally.

*Corresponding authors

Ying Peng: pengy2@mail.sysu.edu.cn

Jinjin Chen: chenjj365@mail.sysu.edu.cn

**Supplementary methods**

**RNA electrophoresis**

The mIL-10 and the ssRNA ladder were mixed with 2 × RNA loading dye, heated at 90 °C for 3 minutes to denature secondary structures, and subsequently placed on ice for 2 minutes. The samples were then electrophoresed on a 1% agarose gel stained with SYBR GOLD and visualized under a UV transilluminator.

**Immunofluorescence**

The spinal cord and DRG tissues were fixed in 4% paraformaldehyde, followed by dehydration in a sucrose gradient ranging from 10% to 30%. The tissues were then sectioned into 15 µm slices using a Leica cryotome. These sections were incubated overnight at 4 °C with primary antibodies specific to GFAP, MAP2, and IBA1, followed by a 2-hour incubation at room temperature with secondary antibodies. Details of the antibodies used can be found in Supplementary Table 4. Immunofluorescence images were captured using a confocal laser scanning microscope (Olympus FV3000) and analyzed with Fiji software.

**Western blotting**

Tissues were homogenized in RIPA lysis buffer containing protease and phosphatase inhibitors. Proteins were separated by SDS‒PAGE and transferred onto PVDF membranes. The membranes were blocked with 5% milk and incubated overnight at 4 °C with primary antibodies targeting GluN1, GluN2A, GluN2B, and tubulin. This was followed by incubation with secondary antibodies. Detailed information about the antibodies used is provided in Supplemental Table 4. Fiji software was used to analyze the densitometry of bands.

**Real-time qPCR**

Total RNA was extracted using TRIzol reagent, and cDNA was synthesized by using the PrimeScript RT Reagent Kit. The cDNA was subjected to qPCR analysis with SYBR Green reaction mix on a LightCycler 384 System (Roche, Basel, Switzerland). Relative mRNA expression levels were calculated using the 2-ΔΔCt method. Primer sequences for Grin1 (encoding GluN1), Grin2a-d (encoding GluN2A-D), and gapdh are provided in Supplementary Table 5.

**ELISA assays**

Cell lysates, along with the cellular supernatants, mouse spinal cord and DRG tissue lysates, mouse serum, and mouse CSF, were collected. Cultured cells and tissues were homogenized in RIPA lysis buffer. The concentrations of IL-10, TNF-α, IL-6, TGF-β, and IL-1β in these samples were measured using ELISA kits (Novus biologicals Co, Ltd.) according to the manufacturer’s protocols.

**Safety evaluation**

After 24 hours of transfection with LPNPs, MA and BV2 cells were incubated with CCK8 reagents for 2 hours, and the absorbance was measured at 450 nm using a microplate reader. C57BL/6J mice were intrathecally injected with P6CIT LPNPs and monitored for one day, one week and one month. The heart, liver, spleen, lung, kidney, spinal cord, and DRG tissues were collected for immunofluorescence analysis and Hematoxylin-Eosin (H&E) staining.

**Figure S1. Representative fluorescence images of MA cells transfected with EGFP mRNA-loaded LNPs.** Scale bar = 100μm.


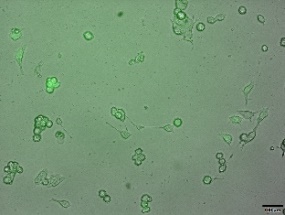

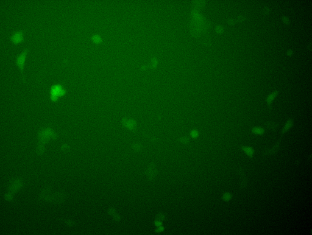

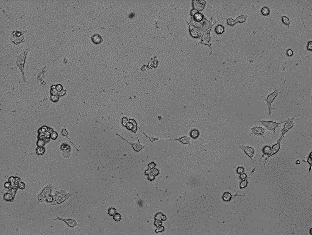

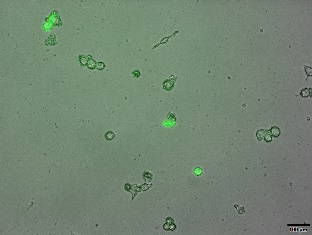

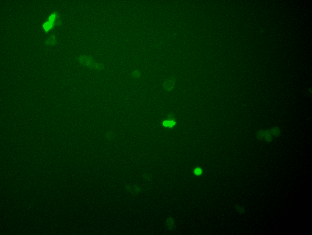

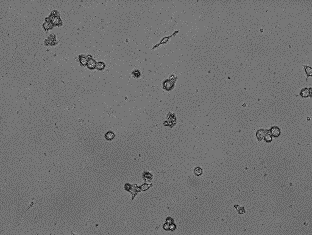

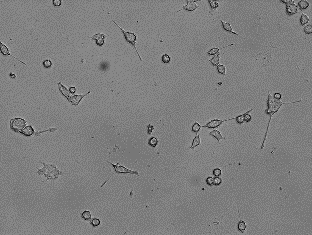

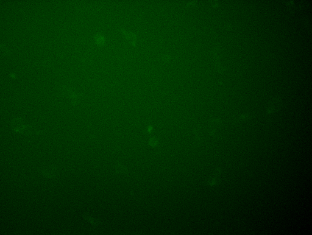

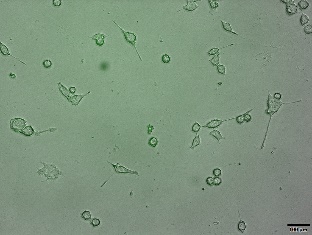

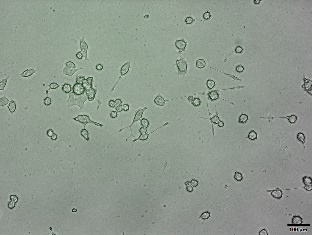

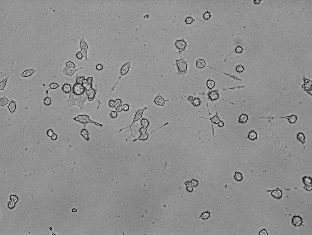

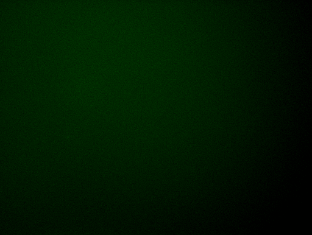


**EGFP**

**Phase contrast**

**Merge**

**NC**

**P6CIT**

**MC3**

**Lipo3000**


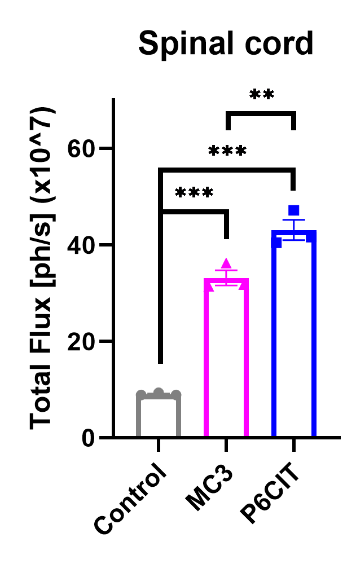

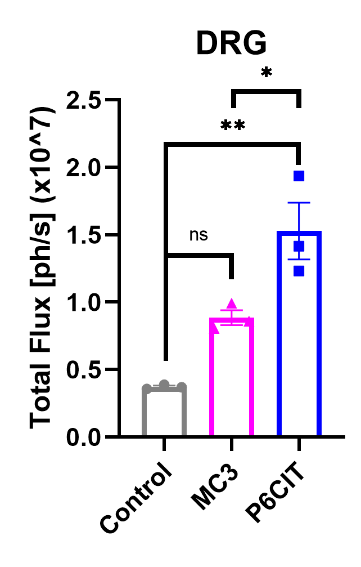


**Figure S2 Quantification of bioluminescence in the spinal cord and DRG of Ai9 mice in different groups.** The data are expressed as the mean ± SEM; **p*<0.05, ***p*< 0.01, ****p*< 0.001; ns, nonsignificant.


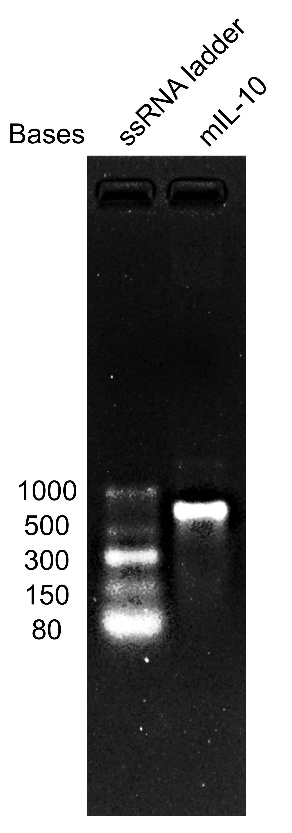


**Figure S3 RNA electrophoresis analysis of the synthesized mIL-10.**


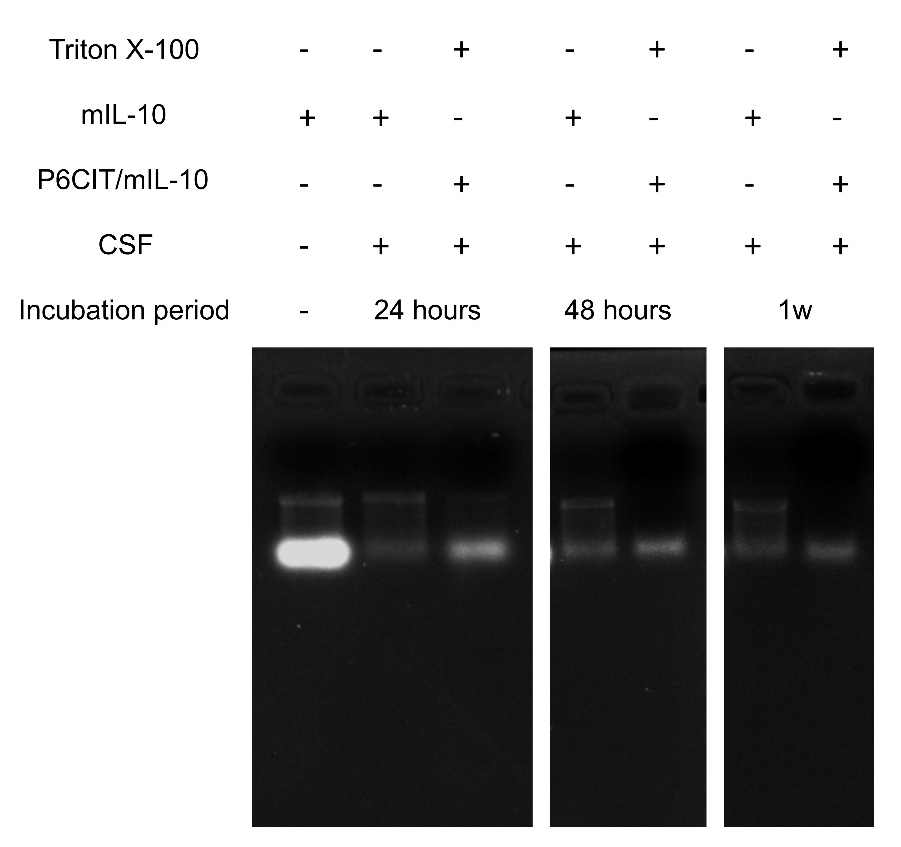


**Figure S4 Stability comparison of free IL-10 mRNA and mIL-10 encapsulated in P6CIT LPNPs after incubation with human CSF for durations of 24 hours, 48 hours, and one week.**


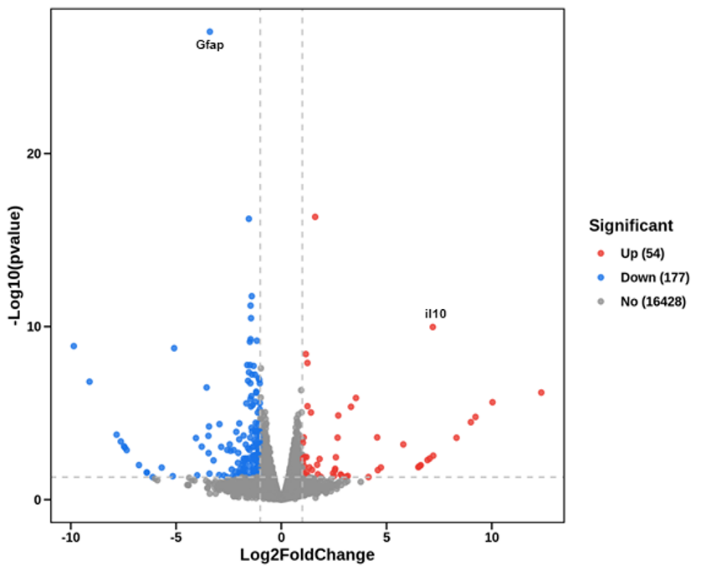


**Figure S5. Volcano map of differentially expressed genes between the PTX + P6CIT/mIL-10 group and the PTX group.**


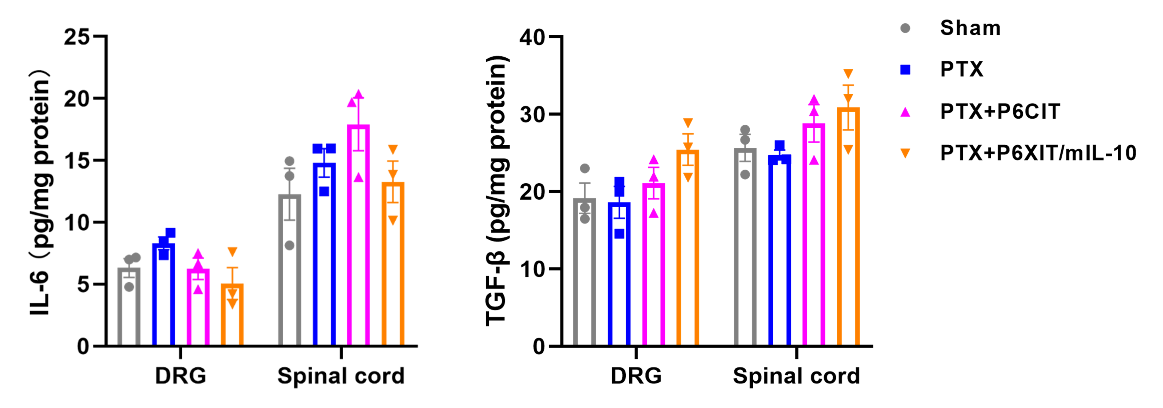


**Figure S6 ELISA analysis of IL-6 and TGF-β levels in the lumbar spinal cord and DRG of mice following different treatments. n=3.**


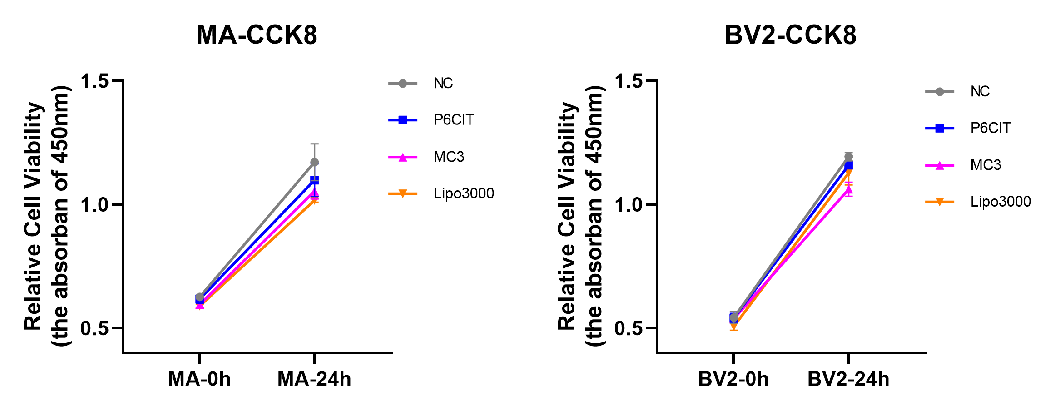


**Figure S7. Cell viability of MA and BV2 cells using CCK-8 assay.** The data are expressed as the mean ± SEM; **p*<0.05, ***p*< 0.01, ****p*< 0.001; ns, nonsignificant. Two-way ANOVA followed by Tukey’s post hoc test.


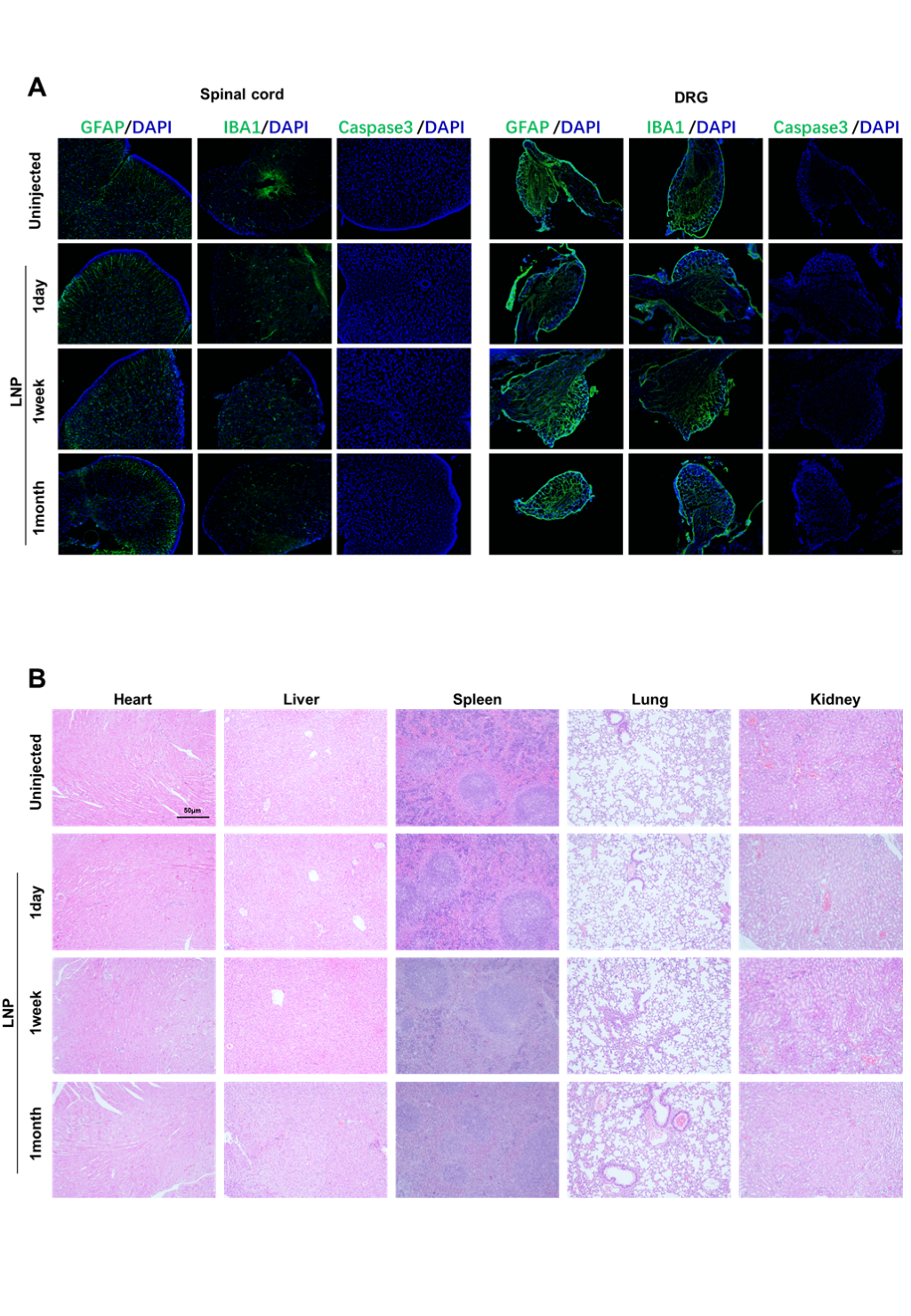


**Figure S8. Biosafety evaluation of mice after intrathecal injection of P6CIT-LNPs. A** Immunohistochemistry for GFAP, IBA1 and caspase 3 in the spinal cord and DRG of C57 mice at one day, one week, or one month after intrathecal injection of P6CIT-LNPs. Scale bar = 100 μm. **B** H&E staining of histological sections of organs including heart, liver, spleen, lung and kidney. Scale bar = 50 μm.

**Table S1. *IL-10* mRNA sequences in this study.**

| Name | Sequence |
| --- | --- |
| Murine *IL-10* mRNA ORF sequence | ATGCCTGGCTCAGCACTGCTATGCTGCCTGCTCTTACTGACTGGCATGAGGATCAGCAGGGGCCAGTACAGCCGGGAAGACAATAACTGCACCCACTTCCCAGTCGGCCAGAGCCACATGCTCCTAGAGCTGCGGACTGCCTTCAGCCAGGTGAAGACTTTCTTTCAAACAAAGGACCAGCTGGACAACATACTGCTAACCGACTCCTTAATGCAGGACTTTAAGGGTTACTTGGGTTGCCAAGCCTTATCGGAAATGATCCAGTTTTACCTGGTAGAAGTGATGCCCCAGGCAGAGAAGCATGGCCCAGAAATCAAGGAGCATTTGAATTCCCTGGGTGAGAAGCTGAAGACCCTCAGGATGCGGCTGAGGCGCTGTCATCGATTTCTCCCCTGTGAAAATAAGAGCAAGGCAGTGGAGCAGGTGAAGAGTGATTTTAATAAGCTCCAAGACCAAGGTGTCTACAAGGCCATGAATGAATTTGACATCTTCATCAACTGCATAGAAGCATACATGATGATCAAAATGAAAAGCTAA |

**Table S2 The hydrodynamic size and PDI of MC3/mCre and** **P6CIT/mCre.**

| Parameters | MC3/mCre | P6CIT/mCre |
| --- | --- | --- |
| Size (nm) | 185.3±3.9 | 159.2±1.0 |
| Zeta potential (mV) | -14.97±0.52 | -3.00±0.14 |

**Table S3 The hydrodynamic size and PDI of P6CIT-LNPs and P6CIT/ mIL-10 12 hours after incubation with human CSF.**

| Parameters | P6CIT + CSF-12h | P6CIT/ mIL-10 + CSF-12h |
| --- | --- | --- |
| Size (nm) | 104.5±0.98 | 116.1±1.54 |
| PDI | 0.287±0.008 | 0.237±0.002 |

**Table S4. Information about antibodies used in this study.**

| Antibody | Dilution | Source | Identifier |
| --- | --- | --- | --- |
| Mouse anti-GFAP | 1:200(IF) | Beyotime | AF0156 |
| Rabbit anti-MAP2 | 1:200(IF) | Proteintech | 17490-1-AP |
| Mouse anti-IBA1 | 1:200 (IF) | Abcam | Ab283319 |
| Rabbit anti-caspase 3 | 1:200 (IF) | Proteintech | 19677-1-AP |
| Rabbit anti-α-tubulin | 1:2000(WB) | Proteintech | HRP-66031 |
| Rabbit anti-GluN1 | 1:1000(WB) | Proteintech | 27676-1-AP |
| Rabbit anti-GluN2A | 1:1000(WB) | Proteintech | 28525-1-AP |
| Rabbit anti-GluN2B | 1:1000(WB) | Proteintech | 21920-1-AP |
| HRP-conjugated goat anti-rabbit secondary antibody | 1:5000(WB) | Proteintech | SA00001-2 |
| Alexa Fluor 488-labeled goat anti-rabbit secondary antibody | 1:200 (IF) | Beyotime | A0423 |
| Alexa Fluor 488-labeled goat anti-mouse secondary antibody | 1:200 (IF) | Beyotime | A0428 |

**Table S5. The primer sequences of targeted genes.**

| Target gene | Primer forward sequences | Primer reverse sequences |
| --- | --- | --- |
| mouse grin1 | TCCAGCTTCAAGAGACGTAGG | CTCTCCCTATGACGGGAACA |
| mouse grin2a | CTGGAAGAGGCAGATTGACC | TCTTCTCGTTGTGGCAGATG |
| mouse grin2b | GCATGCCTACATGGGAAAGT | GTTGAGCACAGCTGCATCAT |
| mouse grin2c | CCTAATGGCAGCACAGAGAGGA | CGGCATCATAGATGAAGGCGTC |
| mouse grin2d | GCTGTCTGGGTGATGATGTTCGTC | TGGATTTCCCAATGGTGAAGGTAGAG |
| mouse gapdh | ACTTCAACAGCAACTCCCACT | TCTCTTGCTCAGTGTCCTTGC |
